# Effectiveness of individualized physiotherapy on pain and functioning compared to a standard exercise protocol in patients presenting with clinical signs of subacromial impingement syndrome. A randomized controlled trial

**DOI:** 10.1186/1471-2474-11-114

**Published:** 2010-06-09

**Authors:** Thilo O Kromer, Rob A de Bie, Caroline HG Bastiaenen

**Affiliations:** 1 Physiotherapiezentrum, Grube 21, 82377 Penzberg, Germany; 2 Department of Epidemiology, Maastricht University, P.O. Box 616, 6200 MD Maastricht, the Netherlands; 3 CAPHRI School for Public Health and Primary Care, Maastricht University, P.O. Box 616, 6200 MD Maastricht, the Netherlands; 4 Centre for Evidence-Based Physiotherapy (CEBP), Maastricht University, P.O. Box 616, 6200 MD Maastricht, the Netherlands

## Abstract

**Background:**

Shoulder impingement syndrome is a common musculoskeletal complaint leading to significant reduction of health and disability. Physiotherapy is often the first choice of treatment although its effectiveness is still under debate. Systematic reviews in this field highlight the need for more high quality trials to investigate the effectiveness of physiotherapy interventions in patients with subacromial impingement syndrome.

**Methods/Design:**

This randomized controlled trial will investigate the effectiveness of individualized physiotherapy in patients presenting with clinical signs and symptoms of subacromial impingement, involving 90 participants aged 18-75. Participants are recruited from outpatient physiotherapy clinics, general practitioners, and orthopaedic surgeons in Germany. Eligible participants will be randomly allocated to either individualized physiotherapy or to a standard exercise protocol using central randomization.

The control group will perform the standard exercise protocol aiming to restore muscular deficits in strength, mobility, and coordination of the rotator cuff and the shoulder girdle muscles to unload the subacromial space during active movements. Participants of the intervention group will perform the standard exercise protocol as a home program, and will additionally be treated with individualized physiotherapy based on clinical examination results, and guided by a decision tree. After the intervention phase both groups will continue their home program for another 7 weeks.

Outcome will be measured at 5 weeks and at 3 and 12 months after inclusion using the shoulder pain and disability index and patients' global impression of change, the generic patient-specific scale, the average weekly pain score, and patient satisfaction with treatment. Additionally, the fear avoidance beliefs questionnaire, the pain catastrophizing scale, and patients' expectancies of treatment effect are assessed. Participants' adherence to the protocol, use of additional treatments for the shoulder, direct and indirect costs, and sick leave due to shoulder complaints will be recorded in a shoulder log-book.

**Discussion:**

To our knowledge this is the first trial comparing individualized physiotherapy based on a defined decision making process to a standardized exercise protocol. Using high-quality methodologies, this trial will add evidence to the limited body of knowledge about the effect of physiotherapy in patients with SIS.

**Trial registration:**

Current Controlled Trials ISRCTN86900354

## Background

Shoulder complaints are one of the most common musculoskeletal complaints seen by health professionals [1-5] with an incidence of 9.5 per 1000 patients presenting to primary care [6] and varying data for point prevalence (6.9% to 26%) [7]. They can lead to a significant reduction of health [6,8], seem to be recurring in nature and do not necessarily resolve over time [9-12]. Thus, shoulder complaints represent a relevant health problem for clinicians, employers and health insurance companies.

Although no standardized diagnostic classification for shoulder complaints exists, most shoulder patients presenting to primary care show clinical signs of subacromial impingement [5,6]. Subacromial impingement syndrome of the shoulder (SIS) occurs due to a mechanical disturbance within the subacromial space and is characterized by pain and functional restrictions mostly during overhead activities in daily life or sporting activities [13]. Potential factors causing or contributing to SIS such as strength, coordination and integrity of the rotator cuff [14-21] and the shoulder girdle muscles [22-26], mechanical or anatomical changes [27-29], hypomobility or instability of the glenohumeral joint or the scapula [16,26,30-33], and the influence of posture [34,35] are discussed in the literature and suggest a multi-factorial aetiology of SIS. Besides the biomedical aspects of SIS, psychological factors such as kinesiophobia or catastrophizing may negatively influence recovery and thus leading to chronic pain and disability [36-41]. The specific diagnosis of SIS is often based on a thorough history and clinical examination; technical examination methods such as MRI or ultrasonography are often not used in first instance [10], also because their diagnostic accuracy is still limited [42-47].

Physiotherapy is often the first choice of treatment for SIS. Between 10 to 30% of all shoulder patients seen in primary care are referred to physiotherapy after initial presentation [5,10,48]. However, the effectiveness of physiotherapy in patients with SIS is still under debate. Conclusions from systematic reviews suggest that physiotherapy-led interventions, combining different methods or techniques, are not more effective than exercises alone except adding manual mobilization to exercises, which seems to be of additional benefit. Most technical treatments such as ultrasound or laser therapy cannot be recommended. However evidence is limited by poor methodological quality, short follow ups and small sample sizes [49-52]. Thus nearly all current systematic reviews emphasize the need for more high quality trials of physiotherapy interventions, especially of combination of treatment techniques.

This trial compares individualized physiotherapy (IP), considering the patients' individual situation, bio-psycho-social aspects, and the WHO-classification of functioning and disability [53] to a standardized exercise protocol (SEP). Physiotherapeutic management is based on clinical examination results and guided by a defined clinical reasoning process, which belongs to one of the basic skills in musculoskeletal physiotherapy [54].

### Aims of the study

a) To investigate the effect of individually planned physiotherapy (IP) on pain and functioning compared to a standard exercise protocol (SEP) in patients presenting with clinical signs of SIS.

b) To compare direct and indirect costs between both interventions.

## Methods

### Study design

**Figure 1:**
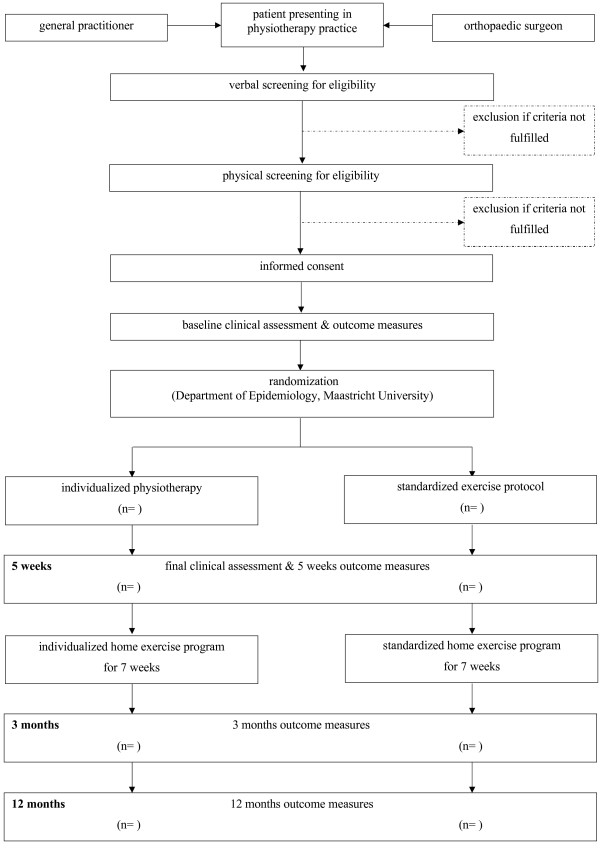
**Trial profile**.

To answer the questions a randomized controlled trial design will be used over a 12 months period. Patients will be randomized after providing informed consent. Randomization and all communication about it is executed and controlled by the Department of Epidemiology, Maastricht University. A flow chart of the trial profile is provided in Figure 1.

### Ethics

Ethical approval for this trial has been granted by the Medical Ethics Committee of the Munich University Hospital, Ludwig-Maximilians-University Munich, Germany.

### Eligibility criteria

Patients presenting to primary care with clinical signs and symptoms indicating SIS will be included in the trial. This concept of focusing on important clinical signs for setting up inclusion criteria for a RCT corresponds well with daily clinical practice.

Inclusion criteria: (1) age between 18 and 75 years, (2) symptoms for more than four weeks, (3) main complaints in the glenohumeral joint region or the proximal arm, (4) presence of one of the following signs indicating SIS: Neer impingement test, Hawkins-Kennedy impingement test, painful arc with active abduction or flexion, (5) pain with one of the following resistance tests: external rotation, internal rotation, abduction, or flexion.

Exclusion criteria: (1) average 24-hours pain of 8/10 or more on a visual numeric rating scale (VNRS), (2) primary scapulothoracic dysfunction due to paresis, (3) diagnosed instability or previous history of dislocation, (4) adhesive capsulitis (frozen shoulder), (5) more than 1/3 restriction of elevation compared to the unaffected side, (6) substantial shoulder weakness or loss of active shoulder function, (7) shoulder surgery in the last 12 months on the involved side, (8) reproduction of symptoms with active or passive cervical movements, (9) neurological involvement with sensory and muscular deficit, (10) inflammatory joint disease (e.g. rheumatoid arthritis), (11) diabetes mellitus, (12) intake of psychotherapeutic drugs, (13) compensation claims, (14) inability to understand written or spoken German.

### Recruitment of participants

The proposed trial will be embedded in the normal daily process of selected physiotherapy clinics in Germany. Participants will be identified by physiotherapy referrals and by research physiotherapists. If a patient agrees to participate, the research therapist will check eligibility criteria. If eligibility is confirmed, informed consent will be asked. Participants will then undergo baseline assessment including some questionnaires and a standardized clinical examination protocol for the shoulder complex, the cervical and upper thoracic spine.

An inclusion period of eighteen months is thought to be sufficient to recruit the number of participants needed for this study.

### Randomization and allocation concealment

After informed consent and baseline assessment participants will be randomized to either SEP or IP using block allocation of six. To guarantee allocation concealment, therapists will be informed about allocation after the participant completed all baseline measurements and gave informed consent, prior to first treatment by the Department of Epidemiology, Maastricht University.

### Interventions

#### Both groups

All participants will undergo a clinical examination process starting with a thorough history taking, followed by a physical examination of the cervical spine, the shoulder girdle, and the shoulder joints. All joints are manually assessed with passive, active, and combined angular movements, and with translatory tests according to the description of Kaltenborn [55], Evjenth and Hamberg [56] or Maitland [57]. Isometric resistance tests are used to judge shoulder strength and pain. Integrity of the rotator cuff is assessed with the external rotation lag sign [58], the lift off test [59], and the hornblowers' sign [60]; involvement of the neural system with upper limb tension tests described by Butler [61]. Contributing factors such as a slouched posture, forward head position, thoracic kyphosis, or protracted shoulders are noted and if necessary also assessed in detail. Results serve as basis for the treatment of participants allocated to the group receiving individualized physiotherapy.

All participants will attend two 20-30 minute contact sessions per week over a 5 week period. Afterwards participants will continue with their home exercises for another 7 weeks. At the beginning of treatment all participants will receive an information booklet containing basic information about anatomy and biomechanics of the shoulder complex, a short description of the aetiology of SIS and the pathology itself, and a brief overview about possible contributing factors to their shoulder pain. The booklet also explains the goals to be achieved with treatment, and it provides general guidelines for behaviour through daily living. Participants will also receive a shoulder log book for documentation of their weekly pain levels, additional treatments or medication, sick leave, and the completion of the home exercise during the intervention phase and during the follow up period. Participants will be requested not to make use of other treatment options and not to change their medication intake during the intervention phase. However, due to ethical considerations the use of analgesics and non-steroidal anti-inflammatory drugs will be permitted and will be recorded in the shoulder log-book.

Treatment will be administered by experienced physiotherapists with an international qualification for manual therapy (IFOMPT standard). All physiotherapists will be trained prior to commencement of the study to guarantee a uniform background and treatment application. A written manual with detailed and comprehensive instructions is given to the therapists. Thoroughness of the application is supported by structured recording forms and check lists, monthly team meetings and audits.

The two groups are as follows:

#### Control group

Participants assigned to the control group will perform a standard exercise protocol (SEP) aiming at restoring muscular deficits in strength, mobility, or coordination of the rotator cuff and the shoulder girdle, unloading the subacromial space, and centering the humeral head in the glenoid fossa during active movements. Thus, the SEP contains mainly strengthening exercises, stretching and mobility exercises, but also exercises to control pain. To set up a high quality protocol, exercises are taken from papers investigating exercises for shoulder rehabilitation [62-79], and exercises specifically addressing deficits in strength, mobility, or coordination revealed in patients with SIS [19,23-25,31,80,81]. Another important criterion for the selection of the exercises was their practicability, their potential for pain provocation, and the possibility to perform all exercises at home with a rubber band. Exercises are subdivided in a "core program" and "additional exercises". A short description of the exercises is provided in Table 1 &2.

**Table 1:** Exercises of the core program.

**No**.	Exercise	Material	Description
C1a	Low row	Pinoband or pulley apparatus with 2 handles	Subject is sitting in front of pinoband, shoulders in 80° forward flexion and neutral rotation; subject performs shoulder extension with elbows flexed.

C1b	High row	Pinoband or pulley apparatus with 2 handles	Subject is sitting in front of pinoband, shoulders in 100° forward flexion and neutral rotation; subject performs shoulder extension with elbows extended.

C2	Shoulder adduction in scapular plane	Pinoband or pulley apparatus with 1 handle	Subject is standing, shoulder in 80° abduction in scapular plane; subject performs shoulder adduction with elbow extended.

C3a	Shoulder external rotation in 0° abduction	Pinoband or pulley apparatus with 1 handle	Subject is standing, with towel between arm and trunk to prevent compensatory shoulder movements, elbow flexed to 90°; subject performs shoulder external rotation.

C3b	Shoulder external rotation in side-lying	Dumbbell	Subject is side-lying, with towel between arm and trunk to prevent compensatory shoulder movements, elbow flexed to 90°; subject performs shoulder external rotation.

C4a	Shoulder internal rotation in 0° abduction	Pinoband or pulley apparatus with 1 handle	Subject is standing, with towel between arm and trunk to prevent compensatory shoulder movements, elbow flexed to 90°; subject performs shoulder internal rotation.

C4b	Shoulder internal rotation in side-lying	Dumbbell	Subject is side-lying, elbow flexed to 90°; subject performs shoulder internal rotation.

C5	Elbow flexion with forearm supination	Pinoband or dumbbell	Subject standing arm at the side, neutral rotation; subject performs elbow flexion/forearm supination.

C6a	Horizontal scapular protraction	Pinoband or pulley apparatus with 2 handles	Subject is standing, elbows flexed to 90°; subject performs shoulder flexion to 80° and elbow extension, then scapular protraction.

C6b	Vertical scapular protraction	Pinoband or dumbbells	Subject lying supine, elbows flexed to 90°; subject performs shoulder flexion to 90° and elbow extension, then scapular protraction.

C7	4-point kneeling scapular protraction	-	Subject in 4-point kneeling position, hands underneath shoulders performs dynamic scapular protraction.

C8	Scapular setting	-	Subject lying prone with arms held by the side in external rotation; subject holds scapulae in depressed and retracted position.

C9	Posterior shoulder stretch	-	Subject is standing, pulling the elbow passively across the body into horizontal adduction with the opposite arm.

C10	Lateral neck stretch	-	Subject is standing, pulling the head into lateral flexion with the opposite arm and is adding the shoulder depression to stretch the ipsilateral neck.

C11	Thoracic spine extension	-	Supine on the floor, hips and knees flexed to 90 degrees, hands supporting the neck, with thoracic kyphosis lying on a towel roll.

**Table 2:** Additional exercises.

No.	Exercise	Material	Description
A1a	Shoulder abduction in scapular plane (scaption)	Pinoband or dumbbell	Subject is standing with feet on the pinoband; subject performs 80° of scaption with elbows slightly flexed and external rotation of the shoulder (thumb up).

A1b	Shoulder flexion	Pinoband or dumbbell	Subject is standing with feet on the pinoband; subject performs 80° of shoulder flexion with elbows slightly flexed and external rotation of the shoulder (thumb up).

A2a	Shoulder press via flexion	Pinoband or dumbbell	Subject is sitting with back supported. Upper arms are in contact with the trunk, elbows are maximally flexed and hands in front of shoulders; subject performs full shoulder flexion and elbow extension.

A2b	Shoulder press via abduction	Pinoband	Subject is sitting with back supported. Upper arms are in contact with the trunk, elbows are maximally flexed and hands next to shoulders; subject performs full shoulder abduction and elbow extension.

A3	Horizontal abduction	Pinoband or pulley apparatus with 1 handle	Subject is sitting in front of pinoband attached in shoulder height, shoulders in 80° forward flexion and external rotation; subject performs horizontal shoulder abduction with nearly extended elbows.

A4	External rotation in supported 80° shoulder flexion	Pinoband or pulley apparatus with 1 handle	Subject is sitting with elbow supported on a table in 80° of shoulder flexion and 90° elbow flexion. Pinoband fixed on the table with other hand; subject performs 90° of external rotation.

A5	Internal rotation in supported 80° shoulder flexion	Pinoband or pulley apparatus with 1 handle	Subject is sitting with elbow supported with the other hand in 80° of shoulder flexion, pinoband fixed in waste height; subject performs 90° of internal rotation.

A6a	Shoulder protraction in kneeling push up position	-	Subject in kneeling push up position, hands underneath shoulders and knees behind hips; subject performs dynamic scapular protraction.

A6b	Shoulder protraction in push up position	-	Subject in push up position; subject performs dynamic scapular protraction.

A6c	Half way push up plus	-	Subject in push up position; subject performs a half way push up with a dynamic scapular protraction at the end of arm extension.

AM1	Internal rotation positioning	-	Subject is placing the hand on the buttock or lower back in a pain-free manner, supported by the other hand.

AP1	Pendulum exercises	Dumbbell or bottle	Subject is standing leaning on a chair or table with the good arm and bending forward at the waist. Relax the shoulder blade and let it drop. Subject performs relaxed forward-backward swings and circle swings using body motion.

AP2	Longitudinal shoulder traction	Pinoband	Subject is standing and slightly side bent with pinoband is wrapped around the wrist and fixed with the feet on the bottom with tension. Subject is relaxing the shoulder to allow for longitudinal traction.

Exercises of the "core program" are introduced and instructed to the patient in detail first, and if patients show good progression, exercises from the pool of "additional exercises" can be added. At home exercises are realized with the help of a PINOFIT rubber band (Pino GmbH, Hamburg, Germany) which allows dynamic resistance and is easy to use. It is available from very light resistance to heavy resistance and allows the therapist to progressively adapt resistance to the physical capacity of the patient. Patients are supervised during their contact sessions; their exercise program is monitored, controlled and adapted if necessary. Physiotherapists are allowed to adapt the SEP individually to each patient with respect to the situation of the patient. Therapists who deliver the treatment for the control group remain blinded to the clinical examination results to prevent inadvertent contamination of the SEP.

#### Intervention group

Participants assigned to the intervention group will perform the SEP as a home program. Additionally this group will receive six to ten session of individualized physiotherapy (IP), based on the findings of the clinical examination and the individual main complaints of the patient. To guarantee a uniform decision making process and to deliver a defined and repeatable way of treatment application a decision tree was developed. The decision tree was previously tested in patients with SIS to improve weaknesses and to test its practicability. It consists of three parts and directs initial treatment applications. The first part of the decision tree addresses predictive signs for a poor treatment outcome such as recurrent episodes of shoulder pain in the past [7,9,11,12], severe pain or long duration of the current episode [82-84], signs indicating a tear of the rotator cuff [85,86], and restriction of external rotation and/or elevation of the shoulder [9,87].

The second part leads the therapist through factors maintaining or contributing to the patients' problem such as general posture, ADL's, working activities and work place setting, leisure and sports activities, and patients' understanding of his problem. The third part guides through the positive findings of the physical examination of the upper quarter (cervical and upper thoracic spine, shoulder and shoulder girdle). Local factors will be treated according to the manual therapy concepts of Maitland [57], Kaltenborn [88], Evjenth and Hamberg [56], or Butler [61].

For further treatment decisions and as an important part of treatment application a defined clinical reassessment process is implemented, adapted from Jones and Rivett [89]. The reassessment process, based on the test-retest-principle, delivers important information about the effect of an applied intervention or technique and thus assists the therapist in further decision making.

#### Main contrast between both groups

The main difference between both groups is that the intervention group additionally receives individualized physiotherapy considering all predictive, local or contributing factors that may maintain or contribute to the patients' problem, identified through clinical examination. Therefore this intervention, combining a shoulder specific exercise programme with a defined decision making process and clinical experience, represents a best practice approach.

### Outcome measures

Selection criteria for the outcome measures used in this study were their reliability and validity in relation to the study population, and also their sensitivity to detect change statistically, whether it is relevant to the patient or clinician or not. Another important criterion was their practical applicability in a clinical setting. The main focus is on pain and functioning. Primary outcome measures will be as follows:

#### 1. Shoulder Pain and Disability Index (SPADI)

The SPADI is a shoulder specific self-reported questionnaire measuring pain and disability in patients with shoulder pain of musculoskeletal origin [90]. It contains 5 items assessing pain and 8 items assessing shoulder function and is easily applicable in daily practice. Each item is scored on a 100 mm visual analogue scale (VAS); the right end of the VAS is defined as "worst pain imaginable/so difficult required help", the left end as "no pain/no difficulty". A score is then calculated out of 100 with higher scores reflecting higher pain/disability levels. The SPADI has shown to be valid and highly responsive in assessing shoulder pain and function [90,91]; it is therefore highly recommended for the use in patients with SIS [92]. The German version of the SPADI also showed an excellent reliability and internal consistency for both, total score and sub-scores. A minimum improvement in the total SPADI score of 11 points will be considered as a minimum clinically important change [93].

#### 2. Patients' global impression of change (PGIC)

Measuring PGIC is a clinically relevant and stable concept for interpreting truly meaningful improvements in pain from the individual perspective [94,95]. It is measured with the help of an ordinal scale with 1-much worsened, 2-slightly worsened, 3-unchanged, 4-slightly better, 5-much better, whereas a rating of "slightly better" will be defined a priori as a clinically important and meaningful difference and therefore as a successful result. According to this definition, the scale is then dichotomized. To test stability of this dichotomization a sensitivity analysis will be conducted.

Secondary outcome measures will be:

#### 1. Generic Patient-Specific Scale (GPSS)

The GPSS is published by Stratford et al. [96] and assesses individual complaints and restrictions in a short and efficient way. It is based on the patient-centred approach, identifying the most problematic areas of functioning.

The GPSS is a reliable and valid tool and also sensitive to detect change over time [97]. Although it is a generic outcome measure, its validity, reliability and sensitivity has been established for different patient groups [96,98,99]. For this study, patients will chose 3 activities they got difficulties with and rate the ability to perform them on an 11-point visual numeric rating scale (VNRS). 10 at the right end of the VNRS is defined as "I can do the chosen function without difficulty", 0 at the left end as "I am unable to do the chosen function". An average score across all activities is calculated. Because the expected change of severely restricted activities is less than the expected change of only mild restrictions, a minimum change of 30% will be considered as a clinically important improvement [95,100].

#### 2. Average weekly pain score

Patients will rate their average weekly pain intensity on an 11-point VNRS. The VNRS is a one-dimensional measure to assess pain intensity. The distance between each number is 10 millimetres; 0 on the left end of the VNRS is defined as "no pain at all", 10 at the right end as "as much pain as I can imagine". An improvement in pain level of 2 points or more was defined as a clinically important and meaningful difference [95,100].

#### 3. Patient satisfaction with treatment

After 5 weeks all patients will rate their satisfaction with treatment on an 11-point visual numeric rating scale (VNRS). 10 at the right end of the VNRS is defined as "completely satisfied", 0 at the left end as "completely dissatisfied".

#### 4. Fear Avoidance Beliefs Questionnaire (FABQ)

It has been shown that fear of movement is an important obstacle to a successful rehabilitation in patients suffering from low back pain. To be able to analyze the influence of fear of movement on treatment outcome in patients with SIS, a modified version of the FABQ is used in this study. The FABQ was developed by Waddell et al. [101] to assess the influence of patients beliefs about physical activity and work on low back pain. The German version of the FABQ shows good psychometric properties and is therefore used in this study [102-105]. The FABQ is a 16-items questionnaire. Each item is scored on a seven-point Likert scale (0 = strongly disagree, 6 = strongly agree). A total score is calculated by summing up the resultant scores. Sub-scores for physical activity and work are calculated, with 7 items assessing beliefs about work (item 6, 7, 9, 10, 11, 12, 15) and 4 items assessing beliefs about physical activity (item 2, 3, 4, 5). Higher scores reflect a higher presence of fear avoidance believes.

#### 5. Pain Catastrophizing Scale (PCS)

Besides fear of movement, catastrophizing may also play an important role in mediating responses to pain, leading to perception of higher pain intensities and therefore influencing treatment outcome negatively [106-108]. The PCS is a multidimensional, reliable and valid 13-item self report measurement tool with a strong association to pain and emotional distress [106,107,109,110]. The PCS has been validated for the German population [111]. It comprises three subscales for rumination (item 8 to 11), magnification (item 6 to 7, 13), and helplessness (item 1 to 5, 12). Each item is rated on a 5-point scale from 0 (not at all) to 4 (all the time). A total score and sub-scores for each subscale are calculated by summing up the ratings for each item within a subscale. In a sample of 86 patients with sustained soft tissue injuries to the neck, shoulders or back including shoulder patients, Sullivan et al. [112] found that catastrophizing was significantly correlated with patients' reported pain intensity, disability and employment status. The rumination subscale was the strongest predictor of pain and disability. Due to a sufficient test-retest stability even over a longer period of time the PCS is a appropriate screening tool for pain catastrophizing [113].

#### 6. Patients' expectancies of treatment outcome

Patients' beliefs about the success of a given treatment may influence treatment outcome; this has been shown by Goossens et al. [114] and Smeets et al. [115] in low back pain patients. For this trial a modified question of the Credibility/Expectancy Questionnaire (CEQ), developed by Deviliya and Borkovecb [116], to measure patients' expectancies is used. The CEQ shows high internal consistency and good test-retest reliability. The question is: "By the end of the therapy period, how much improvement in your limitations due to shoulder pain do you think will occur? The question is scored on an 11-point visual numeric rating scale (VNRS) from 0 (no improvement) to 10 (completely recovered). A higher score will reflect more positive expectancies.

#### 7. Compliance with treatment, direct (health care) and indirect (non-health care) costs

A shoulder log book will be used to obtain the following data: i) Compliance of participants with treatment including the attended treatment visits out of a maximum of ten and the performance of the given home exercises; ii) direct health care costs including physiotherapy, other health provider visits, diagnostic tests, prescriptions and over the counter medication due to shoulder complaints; iii) indirect health care costs including days of sick leave and paid help. The log book is presented in booklet form containing instructions and explanations about the objective of the log book. Log-books will be posted back to the assessor and checked for completion every two months.

Demographic information will also be collected including age, sex, height, weight, profession, sports and leisure activities, medical history, and medication intake. Information about severity and duration of symptoms and previous episodes of shoulder pain are also documented.

### Follow-up evaluation

Patients are assessed at baseline, after completion of the intervention period at 5 weeks, and at 3 and 12 months after inclusion to assess the long term outcome of the intervention. An overview of the outcome measures is given in Table 3.

**Table 3:** Primary and secondary outcome measures

Primary Outcomes	Measurement	Follow up
Shoulder pain and disability index (SPADI)	13 items (5 for pain, 8 for function) scored on a 100 mm visual analogue scale	baseline; 5 weeks; 3, 12 months

Patients' global impression of change	Ordinal scale (1-much worse, 2-slightly worse, 3-no change, 4-slightly better, 5-much better)	5 weeks; 3, 12 months

**Secondary Outcomes**	**Measurement**	**Follow up**

Generic patient-specific scale	11 point visual numeric rating scale (end descriptors of 0 = impossible to do, 10 = no difficulties at all)	baseline; 5 weeks; 3, 12 months

Average weekly pain score	11 point visual numeric rating scale (end descriptors of 0 = no pain, 10 = worst pain possible)	baseline; 5 weeks; 3 months

Patients' satisfaction with treatment	11 point visual numeric rating scale (end descriptors of 0 = completely dissatisfied, 10 = completely satisfied)	5 weeks

Kinesiophobia/Fear avoidance beliefs	Modified fear avoidance beliefs questionnaire (FABQ)	Baseline

Catastrophizing	Pain catastrophizing scale (PCS)	Baseline

Patients' expectancies of treatment effect	One modified question from the Credibility/Expectancy Questionnaire (CEQ)	Baseline

Compliance	Shoulder log book: Attention of treatment sessions and completion and frequency of home exercises	5 weeks; 3, 12 months

Costs	Cost diary: Disease specific healthcare utilization, days of sick leave, drug use, paid help	5 weeks; 3, 12 months

### Sample size

Sample size for this trial is based on an expected difference between groups of a 13 points reduction of the SPADI score. The statistical level of significance was set to alpha = 0.05, statistical power to 80%, and a 15% drop out rate was expected. The assumed standard deviation was set to 20 points based on the results of other studies [117-120]. Power calculations resulted in an estimated sample size of 90 participants (45 per group) to detect a 13 points difference in SPADI score. The minimum clinically important change is set to an 11 points improvement in total SPADI score [93].

### Data analysis

First, descriptive statistics for demographical characteristics of the whole group will be used. Second, descriptive statistics for demographical and clinical characteristics, for baseline results of outcome measures and other potential confounding variables for the intervention group and control group will be used. Differences will be calculated for within-groups results and between-groups comparisons. Results will be calculated according to the "intention-to-treat principle". Between groups-analysis will include differences between baseline and follow up measurements for each clinical outcome measure used, their standard deviations and 95% confidence intervals. Additionally mixed models for the long term follow ups will be used. Influence of baseline differences for outcome measures will be assessed in a multivariable linear regression analysis. Statistical significance is set to p ≤ 0.05, clinical importance will be judged by the lower 95% confidence interval which equals the minimum effect size.

An economic evaluation will compare costs of both treatment options from a societal perspective.

Resources recorded in the shoulder log book will be valued using published prices for medical costs. Costs for over the counter drugs, aids, and paid help will be reported directly by the participants in their log-books.

Productivity costs resulting from loss of paid labor will also be calculated by applying the friction costs method, which limits the period of production loss to the time during the work of the person is not replaced.

Between-group differences in outcomes of mean total costs were analyzed by Student's *t*-tests for unpaired observations.

## Results

Inclusion of participants has started in April 2010. First results are expected in 2012, long term results in 2013.

## Discussion

In order to compare the effectiveness of individualized physiotherapy to a standard exercise protocol a randomized controlled trial design will be used. Our diagnostic and eligibility criteria are purely based on clinical signs and symptoms which correspond very well with clinical practice, and the population usually seen in primary care is well reflected.

Exercises, as a quite simple form of physiotherapeutic treatment, has been shown to be as effective as other physiotherapy-led interventions in the treatment of SIS and are therefore often recommended. However, most investigated forms of physiotherapy-led interventions have been applied as standard protocols without considering individual needs and may be therefore limited in their effect. In this study, treatment of the intervention group, guided by a defined decision making process will address the individual activity and participation restrictions of each patient, predictive signs for a poor outcome, contributing and local factors. To reveal the additional benefit of this intervention, participants of the intervention group will also perform the SEP as a home program.

To strengthen the validity of the trial results, important qualitative methodological factors have been considered in the planning stage of this trial. To prevent selection bias, participants will be randomly allocated to groups via concealed allocation sequence, implemented by a remote clinical trial centre. To minimize performance bias, physiotherapists treating the active control group remain blinded to the results of the clinical examination to prevent contamination of the SEP. Further the statistician remains blinded to group assignment of the participants. Outcome measures used in this trial are easy to apply in daily practice.

To our knowledge this is the first trial comparing individualized physiotherapy led by a defined decision making process to a standard exercise protocol. Results from this trial will add evidence to the limited body of knowledge about the effect of physiotherapy in patients with SIS.

## Competing interests

The authors declare that they have no competing interests.

## Authors' contributions

TOK conceived of the project, led the design and co-ordination of the trial, and wrote the first draft of the manuscript. RADB and CHGB participated in the study design and commented on drafts of this paper. CHGB provided advice for the method section. All authors have reviewed and approved the final manuscript.

## Pre-publication history

The pre-publication history for this paper can be accessed here:

http://www.biomedcentral.com/1471-2474/11/114/prepub
